# Scarlet Fever

**Published:** 1878-10

**Authors:** 


### SCARLET FEVER.

THE "Member of the Massachusetts Medical Society," who recommended BELLADONNA as a sure preventive of *Scarlet Fever*, had better go into a state of retiracy, and never show his diminished head more. No doubt he meant well. But, like a great many good-meaning people before him, he was weak in the upper story. His whole character can be written in a decimal of letters—Good and Green. As green as the grass on Boston Common on the first day of June. He must have been conscious of his being a nobody, of wanting force, lacking in momentum, as he launches out under the flag of the *Massachusetts Medical Society*. If he wanted that venerable name to help him project his idea into general belief, he should have obtained their official sanction to his theory. No, no, gentlemen! if you have anything worth telling, put your own names to it, so that if it is counterfeit, the public may know where to return it. There would be some satisfaction in that, even though there was nothing to redeem the base coin—no other idea, of assets, any better than the one presented for redemption.

Any educated physician, of even moderate observation and study, knows there is no medicine on the face of the earth which will prevent the spread of any epidemic. More, any medicine given steadily during an epidemic, has a natural tendency to act as a cause of the disease, and any person taking it will be more liable to the disease than if they did not take it at all. Wonder if we can make the Ignoramus family understand our meaning, without giving them the headache? It would be inconsistent to do that, for we aim to prevent disease. We will begin by telling them what all doctors know. Or suppose we go to dollars and cents—that is a universal language. A note, to be at par—which we presume is from a Greek word, *para*, which meaning "equal"—is good as gold, dollar for dollar. If it is above par, it is worth more than dollar for dollar. If below par, one dollar is less than a gold dollar. Good health is par. There is nothing better than that. Some people who are well, by shower baths, and tonics, and bitters, seek to get a little better, but they soon die off, usually. In a state of health there is a certain degree of *excitement* in the system. That is, the pulse beats a certain number of times every minute, averaging about sixty-eight. If, during the cholera or any other ordinary epidemic, the pulse does not go below that, and the general system is in good working order, the disease does not attack the person, nor will it as long as he remains in that condition. Nor will he be likely to suffer from the epidemic if he keeps the system steadily above the natural excitement. But the very moment the pulse is below par in rapidity and vigor, that moment is the individual more liable to disease in proportion to the deficiency. A person in good bodily condition, then, is at par, and is not likely to be attacked by any ordinary epidemic disease; and if in that condition he take a glass of brandy, he is less liable still, until the exciting effects of the liquor have subsided, when he falls below the natural standard, just as far as he was a while ago above it, and is in proportion more liable to the disease than if he had taken no brandy at all. Therefore, having commenced taking brandy, he must keep it up day and night, never letting the system go below par, or he is a lost man. Hence it was that men who were always full of liquor escaped cholera in numerous instances, and the report went forth that brandy prevented cholera; thus multitudes were introduced into the wretched habit, and perished in a drunkard's grave.

Let it be taken for granted that a pure, reliable extract of Belladonna could be had anywhere throughout the country. Let it be admitted, also, that it is an infallible preventive in all cases; let us look at the difficulties of its administration. According to the best Homœopathic authority, we must commence giving it for "some time" before the disease attacks the system, or before we are in the infected district. Every one knows that it attacks a family without a single note of warning, as suddenly and as much without intimation as an ærolite falls from the skies. It is more or less prevalent in large cities during the whole year, especially during the winter months; hence, to come within the requisition, Homœopathic families must administer Belladonna to their children every day for six months of the year. We do not hesitate to say that this is an intolerable alternative, and to be repeated winter after winter, as long as a young family are growing up! But let it be admitted that the infinitessimal pills have all the potency claimed for them, when it is declared that under the administration of that medicine, a "rash" appears, something like the scarlet rash, then the administrator is enjoined to cease giving it; but if he does, the system will fall below par, and disease invades it at once.

But when a person takes the scarlet fever, and it does not appear in the form of a rash, it fixes itself on the throat, and we call it *putrid sore throat*—and the child dies. In case the Belladonna, in some instances, does not cause the rash, how know we that it will not cause a spurious sore throat, while the person, looking for the rash all the time, persists in administering the remedy, when its best friends say it should not be longer given.

But we know some systems are many-fold more subject to the influence of a particular medicine than others, to say nothing of constitutions which are not impressive at all by a given drug, and of others to which that same drug acts as a downright poison, of which *ipecac*, one of the mildest of remedies, is a case in point. To these four, the Belladonna is given in equal doses, wholly inefficient in one case, irritating to the over-sensitive, poisonous to a third, while only on a fourth does it exert its legitimate power—to be manifested safely on the skin, or fatally on the throat, no one can tell.

But the real, pure, fresh extract of Belladonna, is "a powerful narcotic poison of tremendous energy." The whole nervous system is prostrated and paralyzed by it. The eyes become blind, the face red, the cheeks swollen, the jaws twitch and snap about, the feeble pulse, cold extremities, with deep delirium or terrible convulsions, preceding the fatal termination. The body at once begins to swell and putrify, livid spots break out upon its surface, while foul blood flows from nose, mouth, and ears. Such are the effects on the human body when given in over doses, or too long, of a medicine which has been so thoughtlessly put into the hands of the families of the land. And when it is recommended, even by Allopaths, to begin with *half a grain*, it can be readily seen how easily a destructive dose might be administered.

So readily does it part with some of its virtues, that it is recommended to be mixed fresh every morning; so that every day, every family who patronizes it, has a new opportunity of committing a fatal mistake as to quantity in weighing it out. But physicians know that medicinal *extracts* soon lose their virtue by exposure, and that as to Belladonna, it is a very rare chance to obtain it out of large cities, because, as soon as it has stood a little while in the city store, it is pushed off to the country, where its qualities cannot be known; for, after it has lost its virtue, it *looks* as well as it ever did. Its warmest friends admit that they have given it a whole season without any effect whatever in consequence of its not being a fresh preparation.

So that, granting it is all that is claimed for it, we see that it is an utter trifling with human life, in consequence of its being, in a measure, unattainable, in its purity, except in and near large cities.

Really, the whole thing seems very much like a speculation, especially as in a long article on the subject in one of our city papers, the name of the village in New England is given, with the assurance that it can certainly be had there in its purity and genuineness, being prepared by a NEW (!!) process. Oh! yes, certainly. But the process being new, it may extract all the anti-scarletine properties out of it—who knows? A pretty good operation, to get all the families in our country to purchase a quarter of a pound of Belladonna a year, costing twenty cents a pound to make it, and selling at two dollars a pound!

But after all, does the daily administration of Belladonna prevent scarlet fever? All that its warmest advocates can say, is, we gave it in a hundred cases, and not one of the persons who took it, took the fever. But there are millions every year who live in the very midst of it, in cities, who neither take the fever nor the medicine. The whole evidence is negative, and consequently is no proof. But even if it did prevent scarlet fever, would it not, after all, be better to let our children take their chances, and be done with it, than to be laboring under so serious a suspense, year after year, and bringing their constitutions under the influence of so potent a remedy every winter? We think they had, because a person is rarely attacked by it a second time—as rarely almost as in measles or small-pox.

People talk a great deal about the old school doctors giving so much medicine. In scarlet fever, they give less than the Homœopaths. In the vast majority of cases of scarlet fever, as it ordinarily presents itself, they give just nothing at all. A cool, well-aired room, clean clothes and bedding, cooling drinks, light food—that's all; and with such a treatment, under the daily visitation of the physician, do nineteen cases out of twenty of scarlet fever, get perfectly well without any untoward results whatever. Therefore, if the scarlet fever is apprehended, secure the daily attendance of your physician, and let all the responsibility rest on him. If you would not tamper with the mending of a five-dollar silver watch, or a dollar Yankee clock, for fear of making matters worse, but send it to the smith at once, so do not tamper with the life of your child by attempting to correct that wonderful machine, which is the highest exhibition of Infinite Power, and as to whose construction you are as utterly ignorant as you are of the nature of the means proposed to be used for its reparation.

